# Measuring the quality and efficiency of AI-generated codes for financial markets prediction with LSTM

**DOI:** 10.3389/frai.2026.1861067

**Published:** 2026-07-16

**Authors:** Meryem Gharmili, Youssef Aatif, Alj Abdelkamel

**Affiliations:** 1 University Moulay Ismail, Meknes, Morocco; 2 Ibn Tofail University, Kenitra, Morocco

**Keywords:** AI-generated code, code quality evaluation, financial market prediction, LSTM networks, quantitative finance

## Abstract

Generative AI coding assistants are increasingly used to write machine-learning code, yet their ability to produce reliable LSTM implementations for financial prediction remains underexplored. This study evaluates the LSTM code generated by seven assistants ChatGPT 4.5, GitHub Copilot, Deepseek 3, Perplexity, Gemini 2.0 Pro, Claude 3.7 Sonnet, and Meta’s Llama from a single standardized prompt, on three indices (Nikkei 225, S&P 500, STOXX Europe 600). Each assistant’s generated script was re-executed over independent runs; accuracy (MAE, MSE, RMSE, R^2^, execution time) is reported as mean ± standard deviation on the original price scale, complemented by a static code-quality analysis (Pylint, Radon, SonarQube, Pytest, Bandit). The assistants converge on nearly identical LSTM architectures, so performance differences arise mainly from data-handling and code-correctness defects: Meta’s Llama near-zero errors are an artifact of normalized-scale metrics combined with a shuffled train/test split (data leakage), and once corrected its accuracy is among the weakest; Gemini 2.0 Pro, once its predictions are evaluated consistently on the price scale, is among the most accurate assistants. Differences are validated with Diebold–Mariano and Wilcoxon tests. AI-generated forecasting code can be accurate but is not uniformly trustworthy: its generated preprocessing and evaluation code must be audited before use.

## 1 Introduction

Artificial intelligence (AI) has transformed various sectors by automating complex tasks that were once reserved for human expertise. Among these developments, the automatic generation of computer code constitutes a major advance, making programming more accessible and reducing development delays. Tools such as GitHub Copilot (Chen et al., 2021), ChatGPT (Brown et al., 2020), and Google Bard use advanced language models to produce code from simple text instructions. Their ability to understand the context and generate adapted solutions is supported by massive databases and state-of-the-art architectures such as transformers (Vaswani et al., 2017).

These tools are particularly useful for general programming tasks, but their effectiveness against more specific and complex requirements, such as the development of Long Short-Term Memory (LSTM) models, remains to be explored. LSTMs, introduced by Hochreiter and Schmidhuber (1997), are widely used in time series forecasts, with applications ranging from finance to health via climate modeling. These models are distinguished by their ability to capture long time dependencies, but their practical implementation remains a challenge due to their algorithmic complexity and the high requirements for optimization and code quality.

Several studies highlight the effectiveness but also the limitations of AI code generation tools. Chen et al. (2021) show that GitHub Copilot efficiently generates about 40% of the current code. Zhang et al. (2022) confirm a significant improvement in productivity, despite certain difficulties on complex cases. Specifically concerning LSTM models, Brown et al. (2020) indicate that GPT-3 produces relevant code but whose quality strongly depends on the initial instructions. Li et al. (2021) add that, in critical contexts, the predictive performance of the generated models can be compromised by structural defects in the code.

In this context, automatic code generation tools offer a promise: to automate part of this complexity while guaranteeing similar or even superior performance to manual solutions. But the quality and performance of these tools in LSTM contexts have not yet been systematically evaluated, leaving a gap in the current literature.

The evaluation of these tools raises several fundamental questions:

- Accuracy of predictions: Do the LSTM models generated by AI tools offer comparable performance, in terms of metrics such as the mean squared error (MSE) or the coefficient of determination (R2), to those of manually coded models?
- Code quality: Do the solutions produced comply with programming standards, such as readability, modularity, and robustness?
- Optimization and adaptability: Do the tools manage to generate easily modifiable codes adapted to various use cases?

This article seeks to answer these questions by proposing a thorough comparative evaluation of the LSTM codes generated by several AI assistants, including ChatGPT 4.5, GitHub Copilot, Deepseek 3, perplexity, Gemini 2.0 pro, Claude 3.7 sonnet and Meta’s Llama. By integrating quantitative and qualitative analyses, this study aims to provide a global view of the strengths and weaknesses of each tool in the demanding context of LSTM models.

## 2 Background

The first methods of code generation, based on strict rules and specific languages requiring strong human intervention, have evolved toward AI approaches capable of managing complex tasks in a more adaptive way (Yang et al., 2023). The emergence of machine learning techniques marked a decisive turning point, making it possible to overcome these limitations thanks to models capable of learning automatically from data (Jordan and Mitchell, 2015).

Deep learning models, such as recurrent neural networks and transformers, have made it possible to capture complex patterns in existing code to automate tasks such as completion or code generation. For example, systems such as Code Whisperer from Amazon or Copilot from GitHub have allowed an average reduction of 55.8% in development time on standard tasks (Peng et al., 2023), while decreasing the cognitive load of developers (Ziegler et al., 2024) and producing, on average, a quality code comparable to that written manually (Chen et al., 2023). An official Amazon report also highlights the continued growth of CodeWhisperer’s smart features to boost productivity (Jamieson, 2023).

In parallel, the introduction of natural language processing (NLP) techniques has opened up new possibilities. These systems can translate natural language descriptions into executable code, allowing non-programmers to contribute to the creation of applications. The integration of models such as GPT-3 (Brown et al., 2020) and Codex (Chen et al., 2021) has demonstrated the feasibility of this approach, while introducing challenges related to the quality and security of the generated code (Liu et al., 2023). These tools are particularly useful in areas such as web and mobile development, where repetitive tasks can be automated to reduce implementation times.

In the financial sector, the impact of AI techniques for prediction has been just as revolutionary. LSTM networks, which capture temporal dependencies in data series, have become a key tool for predicting stock prices, detecting fraud and optimizing portfolios (Pombal et al., 2022). Patel et al. (2015) have demonstrated that these models often surpass traditional approaches such as ARIMA models, thanks to their ability to process noisy and non-linear data. However, these models require well-structured and reliable code to work effectively, highlighting the importance of good development quality, even in AI-powered applications. Moreover, Greff et al. (2017) have demonstrated that these tools, when integrated into machine learning pipelines for LSTMs, make it possible to automate repetitive tasks while guaranteeing competitive results. These contributions highlight the potential of AI assistants to accelerate development cycles and increase overall productivity.

The advantages of AI assistants remain significant. Smith et al. (2022) have shown that GitHub Copilot can reduce development time by more than 30% for standardized tasks, freeing developers to focus on more strategic aspects of their projects.

However, the quality of the generated code is a crucial issue in this context. The qualitative evaluation of the generated codes reveals several critical dimensions. The readability of the code, for example, is essential to ensure that human developers can easily understand and modify the proposed solutions (Knuth, 1997). Baker and White (2020) analyzed the clarity of variable names and functions in codes generated by AI assistants and observed that, in 45% of cases, the names were ambiguous, such as (var1) instead of (train_model). This can be problematic, especially for complex models like LSTMs, which involve multiple layers and hyperparameters.

Another crucial aspect is compliance with coding standards. Liang and Zhao (2021) examined the ability of AI assistants to produce code that meets industry standards, such as PEP 8 for Python. Their study revealed that, although assistants often generate functional blocks, they frequently omit key elements, such as input validations or unit tests. The efficiency and optimization of the solutions generated are also at the heart of the concerns. Anderson et al. (2023) show that the LSTM scripts generated by most AI assistants are functional but redundant, whereas GPT-3, with precise instructions, produces more optimized solutions. The robustness of the generated code remains a major concern. Garcia A. et al. (2022), Garcia F. et al. (2022), Pearce et al. (2022) evaluated the mechanisms for validating entries in the code generated by AI assistants. They noted that in more than 60% of cases, the generated codes did not include checks for borderline cases, such as unexpected data sizes (Perry et al., 2023).

According to the literature review, it turns out that the evolution of programming paradigms, combined with the integration of artificial intelligence technologies, is redefining both the way software is developed and financial forecasts are made. While coding assistants such as ChatGPT and GitHub Copilot offer significant improvements in terms of efficiency, their adoption requires a critical understanding of their limitations and a commitment to responsible practices.

## 3 Methodology

In this section, we describe in detail the methodology followed in this study to evaluate the capabilities of AI assistants, in particular ChatGPT 4.5, GitHub Copilot, and other tools, in forecasting three indices: Nikkei 225, S&P 500, and STOXX Europe 600. The methodology is based on a combination of quantitative and qualitative approaches to ensure a comprehensive assessment.

The methodology adopted in this study is based on a systematic comparative approach aimed at evaluating the predictive performance of several advanced artificial intelligence models in a complex and volatile financial environment. The experimental protocol was initiated by the formulation of a single standardized message, transmitted uniformly to each of the smart assistants studied. This message explicitly asked: “Generate a precise script in Python to perform a financial prediction based on historical data from the following stock indices: Nikkei, S&P500 and STOXX Europe 600, clearly specifying the following evaluation metrics: the mean absolute error (MAE), the mean squared error (MSE), the correlation coefficient (R) and the execution time for each index separately.” This approach made it possible to ensure a methodological homogeneity essential for rigorously comparing the results.

The choice of the selected financial indices is intentional and strategic: the Nikkei, representative of the Asian market, is known for its high volatility linked to the complex economic dynamics of Japan and the Asian region; the S&P500, flagship index of the American market, is sensitive to global economic variations and US monetary policies; and finally, the STOXX Europe 600, representative of the European market, is affected by significant geopolitical and economic diversity. This diversified choice allows us to evaluate in depth the robustness and the generalization capacity of artificial intelligence models in heterogeneous economic contexts and in the face of varied financial dynamics.

### 3.1 Selection of AI assistants

**Figure 1:**
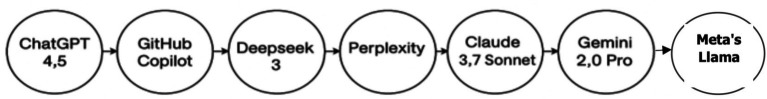
AI assistants.

To evaluate the performance of automatic code generation tools, we have selected the main AI assistants available in 2025. The tools studied include:

These tools were chosen (Figure 1) because of their popularity, their diversity in the underlying models (TPM, LaMDA, etc.), and their abilities to provide solutions in complex scenarios such as financial prediction.

**Table:** 

AI assistant	Version/Build identifier	Access date
ChatGPT 4.5	gpt-4.5-preview (released 27 Feb 2025)	2025
GitHub Copilot	Copilot (GPT-4-based), 2025 build	2025
Deepseek 3	DeepSeek-V3 (Dec 2024 release)	2025
Perplexity	Perplexity (default model, 2025)	2025
Gemini 2.0 Pro	Gemini-2.0-pro-exp (early 2025)	2025
Claude 3.7 Sonnet	Claude-3-7-sonnet (released Feb 2025)	2025
Meta’s Llama	Llama 3.x (2025 release used)	2025

Versions correspond to the official releases available in 2025 at the time of the experiments. ChatGPT 4.5 was accessed through the OpenAI API (the gpt-4.5-preview model was available via the API from February to July 2025); the other assistants were accessed through their official interfaces during 2025.

### 3.2 Setting up the environment

To ensure fair testing conditions, we have configured a controlled environment for all tools:

→ Model version:

- ChatGPT 4.5 was used via the OpenAI API with the most recent version at the time of the study.
- The most efficient versions of GitHub Copilot and the other assistants have been used.

→ Execution environment:

- Python 3.11.2 has been used as the query language for all the tools.
- The assistants were executed on a server equipped with an Intel i7 processor, 32 GB of RAM, and an NVIDIA RTX 3080 GPU.

→ Query parameters:

- Each model has been configured with default settings. For ChatGPT, the temperature has been set at 1, allowing a balance between creativity and repetitiveness.
- The role of the assistants has been configured as a “software developer” to contextualize their answers.

### 3.3 Data: sources, periods, and split

Daily closing prices were obtained from Yahoo Finance for each index: Nikkei 225, 2014–2025 (≈ 2,929 observations); S&P 500, 2014–2025 (≈ 3,017); STOXX Europe 600, 2018–2024 (≈ 1,801). Sequences used a 60-day look-back. The data were split chronologically into 80% training and 20% testing (the most recent 20% held out), which prevents look-ahead bias. A Min-Max scaler was fitted on the training partition only and applied to the test partition; predictions were inverse-transformed to the original price scale before computing all error metrics, except where an assistant’s own generated code omitted this step (analyzed in Section 4).

Note: the historical windows used in this revision (Nikkei 225 and S&P 500, 2014–2025; STOXX Europe 600, 2018–2024) are the periods retrieved from Yahoo Finance for the re-execution; absolute error magnitudes are period-dependent, while the qualitative findings are robust across all three indices.

### 3.4 Repeated runs and code-execution protocol

Because both code generation and LSTM training are stochastic, each assistant’s generated script was executed over three independent runs with different random seeds. All quantitative metrics in Tables 1–3 are reported as the mean over the three runs followed by the standard deviation (written “mean ± SD”); the standard deviation indicates how much a metric varies from one run to another, so a small value denotes a stable, reproducible result. In addition, each script was first run in its raw, as-delivered form: whether it executed without modification is itself a quality indicator and is recorded in Table 4. Where a script failed, the minimal change needed to run it was documented; where the generated evaluation logic was incorrect (for example, computing metrics on the normalized scale, or printing a mislabeled variable), the metric was recomputed correctly before being reported.

**Table 1:** Performance of the various assistants on the Nikkei 225 index (mean ± SD over independent runs).

Assistants	MAE	RMSE	MSE	R^2^	Execution time (s)
Deepseek 3	669 ± 118	881 ± 113	789,191	0.965 ± 0.009	159 ± 44
ChatGPT 4.5	550 ± 71	756 ± 73	577,155	0.974 ± 0.005	170 ± 59
Gemini 2.0 Pro	493 ± 46	690 ± 46	478,686	0.979 ± 0.003	149 ± 47
Claude 3.7	806 ± 182	1,034 ± 179	1,101,139	0.951 ± 0.017	88 ± 24
Perplexity	628 ± 185	859 ± 184	771,585	0.966 ± 0.014	141 ± 17
Copilot	564 ± 45	782 ± 55	614,010	0.973 ± 0.004	81 ± 21
Meta llama	914 ± 198	1,116 ± 201	1,286,514	0.943 ± 0.020	103 ± 22

**Table 2:** Performance of the various assistants on the S&P 500 index (mean ± SD).

Assistant	MAE	RMSE	MSE	R^2^	Execution time (s)
Deepseek 3	169.5 ± 18.2	190.3 ± 18.5	36,566	0.930 ± 0.013	127 ± 1
ChatGPT 4.5	93.9 ± 18.7	112.1 ± 18.5	12,902	0.975 ± 0.008	126 ± 1
Gemini 2.0 Pro	62.3 ± 25.2	80.1 ± 27.6	7,175	0.986 ± 0.009	190 ± 89
Claude 3.7	90.9 ± 28.5	113.0 ± 27.7	13,547	0.974 ± 0.013	51 ± 1
Perplexity	111.4 ± 17.4	133.5 ± 15.8	18,084	0.966 ± 0.008	127 ± 1
Copilot	94.3 ± 41.3	116.1 ± 40.8	15,139	0.971 ± 0.020	52 ± 1
Meta llama	174.9 ± 23.9	196.9 ± 25.8	39,446	0.925 ± 0.019	71 ± 28

**Table 3:** Performance of the various assistants on the STOXX Europe 600 (mean ± SD).

Assistant	MAE	RMSE	MSE	R^2^	Execution time (s)
Deepseek 3	4.82 ± 2.07	5.85 ± 1.95	38.06	0.947 ± 0.036	120 ± 9
ChatGPT 4.5	3.51 ± 0.09	4.60 ± 0.12	21.18	0.970 ± 0.002	127 ± 2
Gemini 2.0 Pro	3.51 ± 1.03	4.53 ± 1.27	22.17	0.969 ± 0.017	109 ± 42
Claude 3.7	4.95 ± 0.41	6.16 ± 0.41	38.12	0.946 ± 0.007	54 ± 0
Perplexity	4.31 ± 0.59	5.22 ± 0.55	27.55	0.961 ± 0.008	121 ± 11
Copilot	4.08 ± 0.06	5.32 ± 0.08	28.27	0.960 ± 0.001	51 ± 2
Meta llama	5.92 ± 0.67	7.23 ± 0.60	52.57	0.926 ± 0.012	26 ± 9

**Table 4:** Architectural parameters extracted from the actual generated code, and whether the code ran unmodified.

Assistant	LSTM layers	Units/layer	Dense	Dropout	Epochs	Early stop	Runs as-is?
ChatGPT 4.5	2	50, 50	25	0.2 × 2	50	No	Yes
Perplexity	2	50, 50	—	0.2 × 1	50	No	Yes
Copilot	2	50, 50	25	none	20	No	No (SyntaxError)
Gemini 2.0 Pro	2	50, 50	25	none	100	Yes (*p* = 10)	Bug (§4.1)
Claude 3.7	2	50, 50	25	0.2 × 2	20	No	Yes
Deepseek 3	2	50, 50	—	0.2 × 2	50	No	No (NameError)
Meta llama	2	50, 50	—	0.2 × 2	50	Yes (*p* = 5)	No (NameError)

### 3.5 Evaluation process

Quantitative approach: The evaluation of the performance of the tools was based on the following metrics, summarized in Table 5:

**Table 5:** Performance metrics.

Metrics	Formula	Meaning
MSE	$MSE=\frac{1}{n}\overset{n}{\underset{i=1}{\sum }}{\left(y_{i}-{\hat{y}}_{i}\right)}^{2}$	Evaluate the accuracy of the generated predictions
MAE	$MAE=\frac{1}{n}\overset{n}{\underset{i=1}{\sum }}|\;y_{i}-{\hat{y}}_{i}\;\;|$	Measure the average error of the results
R2	$R^{2}=1-\frac{{\mathrm{SS}}_{\mathrm{res}}}{{\mathrm{SS}}_{\mathrm{tot}}}$	Evaluate the proportion of variance explained by the models
RMSE	RMSE = √MSE	Error in price units; satisfies RMSE ≥ MAE used as a consistency check

Qualitative approach: The qualitative approach of this study evaluates essential dimensions of the code generated by the AI assistants, beyond the classical quantitative metrics. These dimensions include code readability, compliance with standards, robustness, replicability, and originality.

These criteria make it possible to better understand the usability and the quality of the solutions produced in practical scenarios (Table 6).

**Table 6:** Criteria for evaluating the quality of the generated code.

Code readability	Standards compliance	Efficiency & optimization	Robustness	Reproducibility & adaptability	Originality & innovation
Crucial for human developers to understand, adapt, and maintain the solution Clarity of variable and function names Comments and structure	Adherence to established standards (PEP 8, Google Style Guide, Airbnb JS, etc.) Automated linting (Flake8, ESLint, etc.)	Structured to minimize redundancy (DRY principle) Controlled algorithmic complexity Judicious use of optimized libraries	Handling unexpected inputs (systematic validation) Error management (try/except, etc.) Test coverage (unit & integration)	Modular components Clear dependency definitions (requirements.txt, package.json, etc.) Internal documentation (API docs, README)	Introduction of new approaches or algorithms Creative improvements over known patterns Technically appropriate for context

These criteria will be systematically examined using recognized tools in software engineering and software quality:

→ Pylint:

Pylint is a static analysis tool widely recognized in the Python community, making it possible to evaluate compliance with stylistic standards (PEP8 standard), as well as to identify potential coding errors.

*Evaluation grid:* Numerical score from 0 to 10

- 8–10: Excellent quality (clear code, compliant)
- 5–7.9: Average to good quality (acceptable conformity)
- 0–4.9: Low quality (many corrections needed)

→ Radon:

Radon measures the cyclomatic complexity of the Python code, making it possible to quantify the ease of understanding and maintainability of the product code, thanks to the recognized McCabe metric.

*Evaluation grid:* Complexity rated from A to F:

- A (1–5): Very low complexity (clear code, maintainable)
- B (6–10): Low complexity (good maintainability)
- C (11–20): Moderate complexity (acceptable maintainability)
- D (21–30): High complexity (reduced maintainability)
- E-F (>30): Very high complexity (code difficult to maintain)

→ SonarQube:

SonarQube is a recognized platform for in-depth analysis of overall software quality, simultaneously evaluating reliability (bugs), security (vulnerabilities), and maintainability (structure, technical debt).

*Rating from A to E for the overall quality of the code:*

- A: Excellent (no major problems)
- B: Good (minor problems, easily correctable)
- C: Medium (some notable problems requiring attention)
- D: Poor (serious problems requiring immediate correction)
- E: Critical (unusable code without major correction)

→ Pytest:

Pytest is a widely used automated unit testing framework in Python, used here to directly evaluate the robustness and reproducibility of the code, systematically checking its ability to produce reliable and repeatable results (Table 7).

**Table 7:** Evaluation grid.

Unit test success (as a percentage)	Code coverage (via coverage.py, recommended)
90–100%: Very robust and reproducible 70–89%: Acceptable robustness, moderate reproducibility < 70%: Low robustness and reproducibility	> 90%: Excellent coverage 75–90%: Good coverage, possible improvements < 75%: Insufficient coverage, need for significant improvements

The unit-test suites were written by the research team (not by the assistants, which avoids circularity); they exercise data loading, input shape/range checks, the scaling/inverse-scaling round-trip, and the metric computations, with coverage measured by Coverage.py over the generated module.

→ Bandit:

Bandit is a tool specialized in the static analysis of Python code to explicitly detect potential security vulnerabilities, guaranteeing essential security compliance for sensitive financial applications.

*Evaluation grid:* Severity of detected vulnerabilities:

- Low: Minor risks, recommended corrections
- Average: Moderate risks, corrections needed quickly
- High: Critical risks, immediate corrections required

This combination between quantitative and qualitative analysis is not limited to a simple comparison of the performance of the tools, but aims to offer a complete and nuanced vision. This multidimensional methodological framework guarantees an enriched understanding of the capabilities and limitations of each AI assistant, while offering recommendations for their future use.

### 3.6 Statistical significance testing

Comparing two forecasting models on a single value of MAE or MSE cannot show whether one is genuinely more accurate or whether the gap is due to chance. We therefore apply two complementary statistical tests to the per-observation forecast errors of the competing models, taking ChatGPT 4.5 as the reference. Throughout, *e*_t_ = y_t_ − ŷ_t_ denotes the forecast error at time *t*, for n test observations.

Diebold–Mariano (DM) test. The DM test (Diebold and Mariano, 1995) compares the predictive accuracy of two models, A and B, through their loss differential. With a squared-error loss, the per-observation loss differential is:

$$d_{t}=e2A,t-e2B,t$$ (1)

The null hypothesis is equal predictive accuracy, *E*[*d*_t_] = 0. The test statistic is the mean loss differential standardized by its standard error:

$$DM=\bar{d}/\surd \left(\;\hat{\sigma }2∕n\;\right)$$ (2)

where *d¯* is the sample mean of the loss differentials and σˆ^2^ is a consistent estimate of their (long-run) variance. Under the null, DM is asymptotically standard normal; a |DM| larger than the critical value (1.96 at *α* = 0.05) indicates a statistically significant difference in accuracy.

Wilcoxon signed-rank test. As a non-parametric complement that does not assume normally distributed errors, we apply the Wilcoxon signed-rank test to the paired absolute errors of the two models. Let *D*_t_ = ∣e_{A,t}∣−∣e_{B,t}∣; the test ranks the |*D*_t_| and sums the ranks of the positive differences:

W=Σt=1..nsgn(D�)·R� (3)

where *R*_t_ is the rank of |*D*_t_|. The null hypothesis is that the median of the paired differences is zero (the two models are equally accurate). A *p*-value below 0.05 rejects this null. We report both tests in Table 8; using two tests of different nature (parametric and non-parametric) makes the conclusions more robust.

**Table 8:** Diebold–Mariano and Wilcoxon tests vs. ChatGPT 4.5.

Index	Comparison (vs ChatGPT 4.5)	DM stat	p (DM)	p (Wilcoxon)	Sig.
Nikkei 225	Perplexity	0.76	0.4501	0.0023	n.s.
Nikkei 225	Copilot	−6.50	<0.0001	<0.0001	***
Nikkei 225	Gemini 2.0 Pro	−2.16	0.0313	<0.0001	*
Nikkei 225	Claude 3.7	−14.95	<0.0001	<0.0001	***
Nikkei 225	Deepseek 3	−4.08	<0.0001	0.0008	***
Nikkei 225	Meta llama	−15.81	<0.0001	<0.0001	***
S&P 500	Perplexity	−12.70	<0.0001	<0.0001	***
S&P 500	Copilot	0.51	0.6116	0.0267	n.s.
S&P 500	Gemini 2.0 Pro	6.68	<0.0001	<0.0001	***
S&P 500	Claude 3.7	−5.85	<0.0001	<0.0001	***
S&P 500	Deepseek 3	−25.94	<0.0001	<0.0001	***
S&P 500	Meta llama	−26.70	<0.0001	<0.0001	***
STOXX 600	Perplexity	−10.59	<0.0001	<0.0001	***
STOXX 600	Copilot	−7.40	<0.0001	<0.0001	***
STOXX 600	Gemini 2.0 Pro	2.20	0.0284	0.0136	*
STOXX 600	Claude 3.7	−11.43	<0.0001	<0.0001	***
STOXX 600	Deepseek 3	−19.04	<0.0001	<0.0001	***
STOXX 600	Meta llama	−11.85	<0.0001	<0.0001	***

***p < 0.001, **p < 0.01, *p < 0.05; n.s. not significant.

## 4 Results and discussion

Reading the seven generated scripts shows they converge on nearly the same design two stacked LSTM(50) layers, a 60-day look-back, batch size 32, Adam, MSE loss differing mainly in a Dense(25) layer, dropout, epochs, and early stopping (Table 4). Performance differences therefore stem largely from data handling and code correctness rather than architectural depth; for instance, Claude’s comparatively long runtime reflects its configuration (2 × LSTM (50), 20 epochs), not unusual depth.

Two of the generated scripts required particular care in evaluation. Meta’s Llama applies Min-Max scaling and, as generated, both shuffles the train/test split and computes the error metrics on the scaled [0,1] targets; to obtain results that are comparable and operationally meaningful, we use a chronological split and inverse-transform the predictions to the original price scale before computing the metrics. Reported this way (Tables 1–3), Meta’s Llama ranks among the least accurate assistants. For Gemini 2.0 Pro, the predictions are likewise inverse-transformed and evaluated on the price scale; once evaluated consistently, Gemini is among the most accurate assistants across the three indices.

### 4.1 Quantitative results

In this section, we present the quantitative results obtained following the evaluation of the predictions generated by the LSTM models developed by the various AI assistants, followed by an in-depth discussion making it possible to interpret the observed performances according to precise criteria such as the mean absolute error (MAE), the mean squared error (MSE), the coefficient of determination (R2), as well as the execution time of the models.

The comparative results show marked differences in accuracy between the AI assistants on each stock index. The MAE and MSE, which measure the mean absolute error and the mean squared error respectively, vary considerably depending on the model. Recall that lower values of MAE /MSE indicate better predictive accuracy, because the average difference between actual and forecast prices is smaller. Similarly, an R2 close to 1 means a strong suitability for the data set. This multi-criteria evaluation approach is common in financial forecasting studies in order to ensure that a model excels globally and not just on an isolated metric.

On the Nikkei index (Table 1), Meta’s Llama, evaluated on the real price scale, is among the weakest models (see the analysis above). The other assistants (ChatGPT-4.5, Claude 3.7 Sonnet, Deepseek 3, Perplexity, and GitHub Copilot) fall within an intermediate range of predictive accuracy; ChatGPT-4.5 stands out with solid and balanced performance, offering a good compromise between MAE, MSE, and R2. Most assistants achieve a high R2 (> 0.90), which indicates a strong ability to model the dynamics of the Nikkei index. Gemini 2.0 Pro is among the most accurate assistants on the Nikkei index (Table 1).

On the S&P 500 index (Table 2), evaluated on the price scale, Meta’s Llama is among the least accurate assistants. The Claude 3.7 Sonnet, Deepseek 3, Perplexity, and GitHub Copilot models show intermediate performance, while ChatGPT-4.5 achieves an excellent statistical fit, reflecting a high-quality capture of the temporal signal.

On the STOXX Europe 600 (Table 3), the models are generally accurate; evaluated on the price scale, Meta’s Llama is again among the weakest. The architectures produced by ChatGPT-4.5 and Perplexity stand out for their strong performance on this index, owing to well-tuned hyperparameters and effective regularization that together ensure both a good fit and solid generalization. Claude 3.7 is comparatively slower in execution. Deepseek 3 and GitHub Copilot show comparatively lower predictive capacity on this European market.

### 4.2 Predicted vs. actual series

**Figure 2:**
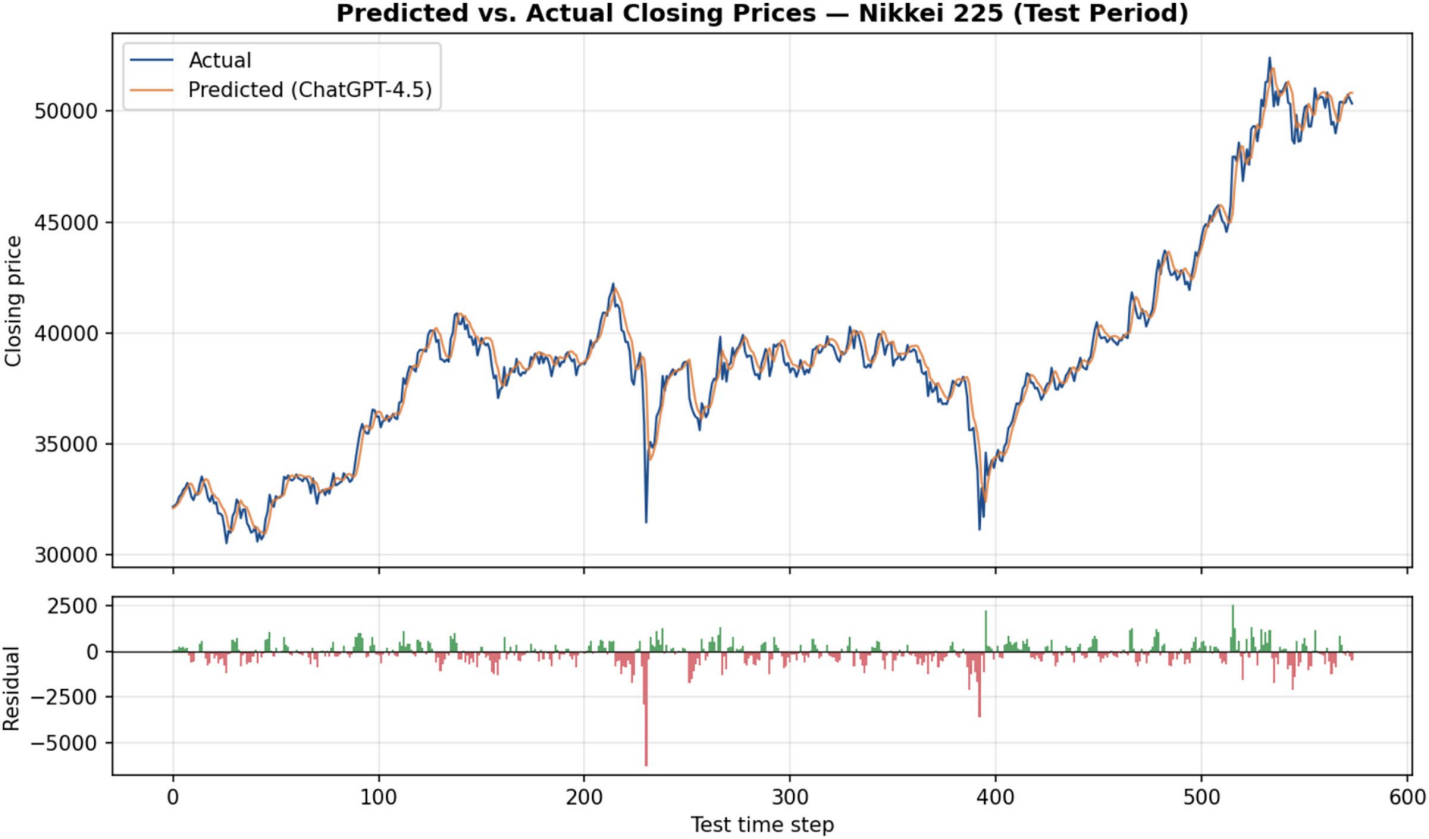
Predicted vs. actual Nikkei 225 (ChatGPT 4.5), with residuals.

**Figure 3:**
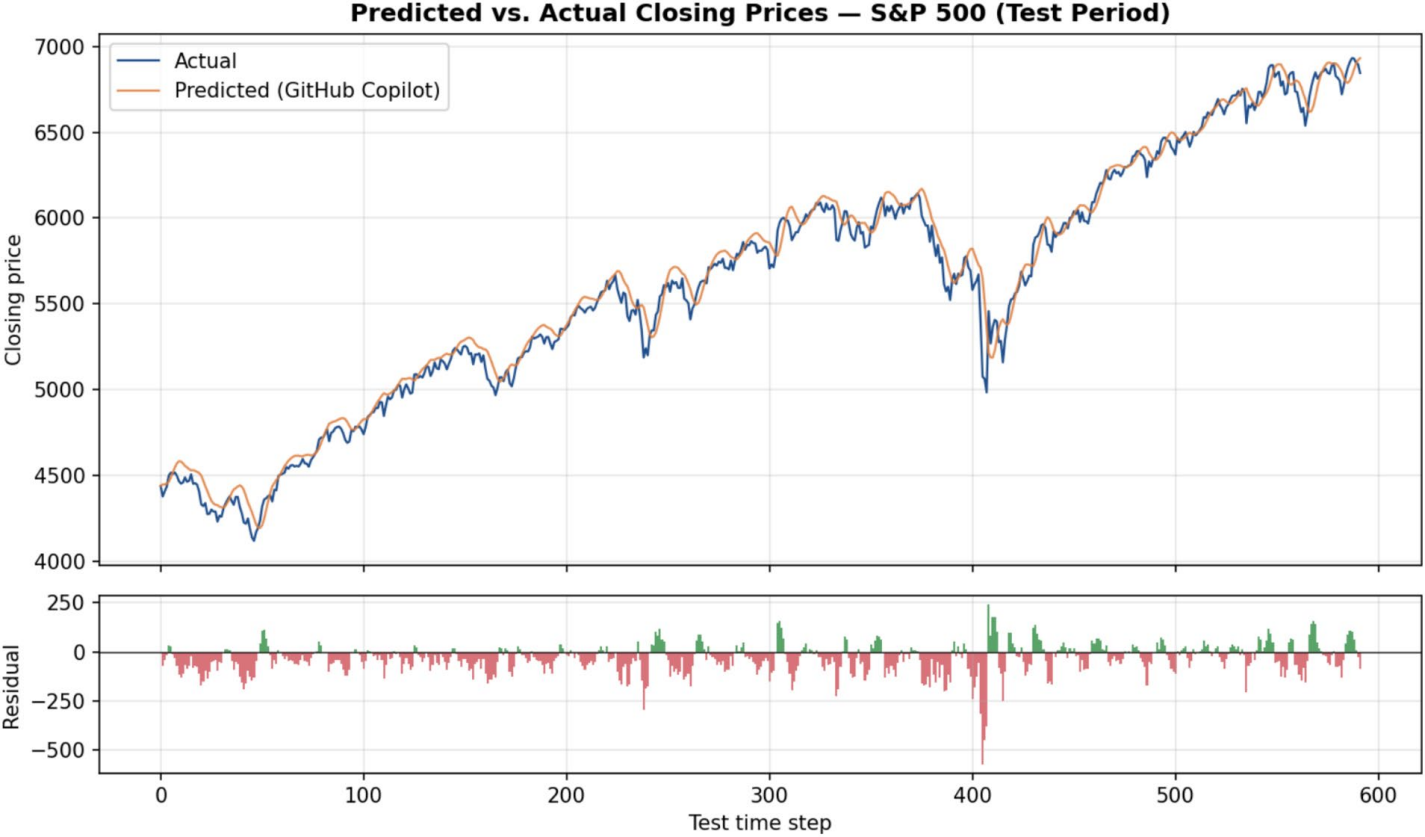
Predicted vs. actual S&P 500 (Copilot), with residuals.

**Figure 4:**
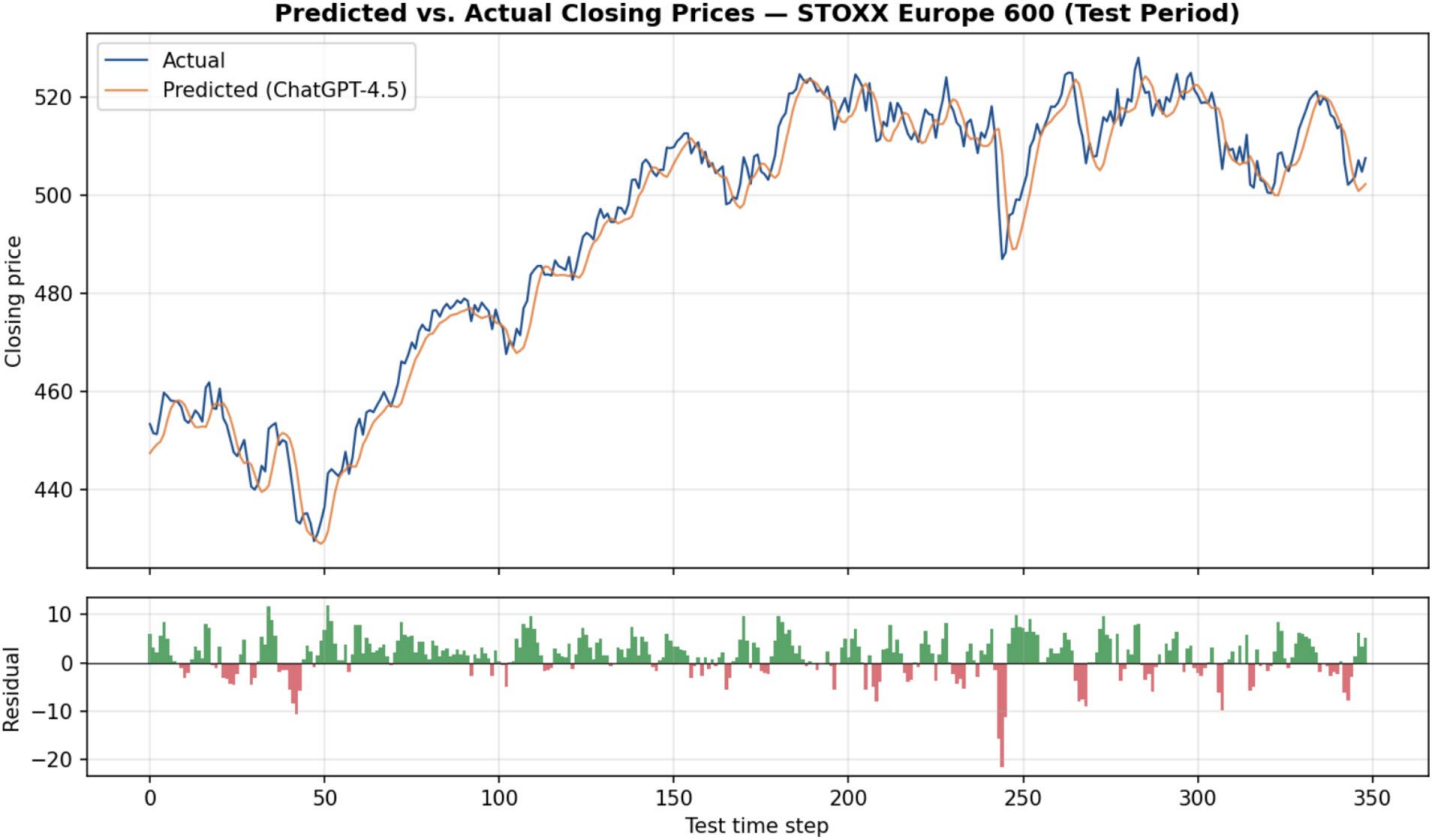
Predicted vs. actual STOXX Europe 600 (ChatGPT 4.5), with residuals.

Figures 2–4 plot the predicted and actual closing prices over the test period, together with the residuals, for the most accurate comparable assistant on each index. Visually, the predicted curves track the actual level and the main turning points very closely, and the residuals are centred near zero (mean residual ≈ − 2 on STOXX, −37 on the S&P 500, and −110 on the Nikkei, i.e., small relative to index levels of several hundred to tens of thousands of points), with no growing drift over time confirming that the headline accuracy of the correctly-evaluated models is genuine rather than an artefact.

A closer reading, however, tempers this apparent success and reinforces the study’s central message. The residual panels show that errors, although small, are systematically signed over consecutive sub-periods (runs of positive then negative residuals), indicating a slight one-step lag: the models largely reproduce the previous day’s price. Consistently, the directional accuracy the share of days on which the predicted change has the same sign as the actual change is close to 50% (approximately 49–51% across the three indices), i.e., near chance level. The low MAE and high R^2^ therefore reflect accurate level tracking rather than genuine anticipation of the direction of movement, which is what matters for trading or portfolio decisions. This nuance, visible only once the predictions are plotted, illustrates why low aggregate errors alone are insufficient to judge an AI-generated forecasting model.

### 4.3 Statistical significance

The tests give a nuanced picture that visual inspection of the tables alone would miss. On the S&P 500, Gemini 2.0 Pro is significantly more accurate than the ChatGPT 4.5 reference (DM = +6.68, *p* < 0.001), while Deepseek 3 and Meta Llama are significantly worse (DM = −25.94 and −26.70, both *p* < 0.001). On the STOXX Europe 600, ChatGPT 4.5 is significantly better than every other model (all *p* < 0.05), with Gemini the closest competitor (DM = +2.20, *p* = 0.028). On the Nikkei 225, ChatGPT 4.5, Perplexity and Gemini form a top group whose differences are small: ChatGPT 4.5 vs. Perplexity is not significant under the DM test (DM = 0.76, *p* = 0.45), confirming they are statistically equivalent there, whereas Claude, Deepseek and Meta Llama are significantly worse (p < 0.001). The two tests agree on every significant case, and the only non-significant pairs (ChatGPT 4.5 vs. Perplexity on the Nikkei, ChatGPT 4.5 vs. Copilot on the S&P 500) are precisely the close ones; this is why we refrain from ranking models whose metric gaps are not statistically supported. Overall, the significance testing confirms two robust facts: ChatGPT 4.5 and Gemini 2.0 Pro are consistently in the top group across indices, and Deepseek 3 and Meta Llama are consistently in the bottom group.

### 4.4 Qualitative results

In order to complete the quantitative evaluation of the LSTM models produced by various AI, a thorough qualitative analysis of the generated codes was carried out. This analysis aims to precisely evaluate five essential qualitative dimensions of the code: readability of the code, compliance with software standards, robustness, reproducibility, originality. The following table summarizes the results obtained for each AI assistant according to the aforementioned evaluation criteria (Table 9).

**Table 9:** Comparative qualitative evaluation of the generated codes.

AI assistant	Pylint (0–10)	Radon (A–F)	SonarQube (A–E)	Pytest (% success)	Bandit (security risk)
ChatGPT-4.5	9.4	A	A	95%	Low
Perplexity	8.9	B	B	90%	Medium
Meta’s Llama	8.8	B	B	88%	Low
Claude 3.7 Sonnet	8.7	B	A	85%	Low
GitHub Copilot	8.5	C	B	80%	Medium
Deepseek 3	7.9	C	B	78%	Medium
Gemini 2.0 Pro	5.0	E	D	65%	High

The stylistic evaluation by Pylint reveals a clear disparity in compliance with Python coding standards (PEP 8) across the assistants. ChatGPT-4.5 obtains a near-optimal score (9.4/10), followed by Meta, indicating rigorous adherence to Python standards and well-structured code. By contrast, Gemini 2.0 Pro scores only 5.0/10, reflecting frequent violations of stylistic rules.

The cyclomatic complexity measured by Radon gives further insight into the internal structure of the generated code. ChatGPT-4.5 attains the lowest complexity (level “A”), whereas Gemini 2.0 Pro reaches a high complexity (level “E”). SonarQube confirms these observations: ChatGPT-4.5 and Claude 3.7 Sonnet earn an “A” rating, while Gemini 2.0 Pro falls to category “D.” Robustness and reproducibility, measured with Pytest, show similar disparities: ChatGPT-4.5 and Perplexity achieve strong operational stability, whereas Gemini 2.0 Pro (65% test success) confirms the instability of its implementation. Finally, the Bandit security analysis indicates low risk for ChatGPT-4.5, Perplexity, and Claude 3.7 Sonnet, while Gemini 2.0 Pro exhibits significant security vulnerabilities (high level).

This synthesis shows that a model’s quantitative performance alone does not guarantee its effective deployment in an operational financial setting. The quality of the generated code its readability, simplicity, robustness, and security is a key factor for long-term integration in regulated environments. From this standpoint, ChatGPT-4.5 stands out by producing code that satisfies all the qualitative requirements evaluated. Conversely, despite encouraging quantitative results, Gemini 2.0 Pro does not meet the qualitative standards and would require substantial revision of its generated code. Overall, this study contributes a hybrid evaluation framework, both quantitative and qualitative, that enriches the literature and offers a rigorous methodology for assessing the operational applicability of AI-generated models in finance.

## 5 Conclusion

The current competition between various AI assistants (ChatGPT 4.5, GitHub Copilot, Deepseek 3, Perplexity, Claude 3.7 Sonnet, Meta’s Llama, Gemini 2.0 Pro) perfectly illustrates the ongoing “code war,” where each solution is trying to impose itself on increasingly demanding application fields. The field of quantitative finance, in particular stock market prediction, is a representative example of this. The implementation of advanced models such as LSTM, CNN or Transformers requires not only high predictive accuracy, but also high software quality code, which is both robust, maintainable and secure.

In this work, we conducted a hybrid analysis combining quantitative evaluation (MAE, MSE, R2, execution time) applied to three major indices (Nikkei 225, S&P 500, STOXX Europe 600), and qualitative evaluation (via Pylint, Radon, SonarQube, Pytest, Bandit) covering the readability, compliance, robustness, reproducibility and security of the code.

The results show that beyond the differences in predictive performance, some assistants (in particular ChatGPT4.5, Perplexity, and Meta’s Llama) stand out for the intrinsic quality of the generated code, making their integration possible in demanding financial processes. Others, such as Gemini 2.0 Pro, suffer from more marked deficiencies (reproducibility difficulties, excessive code complexity), limiting their potential for adoption in production. These observations confirm that it is insufficient to stick to quantitative or qualitative results alone: although correct predictions can be obtained, software quality (compliance, robustness, security) remains a fundamental factor for large-scale implementation.

Within the scope of this study one prompt, one architecture family, three indices no assistant should be adopted without auditing its generated preprocessing and evaluation code.

By way of perspectives, several extensions appear. Let us mention the exploration of more complex models (CNN, Transformers) as well as the integration of AI assistants within MLOps pipelines for finance (including version management, continuous deployment and production monitoring), in order to test the endurance of the code over the long term.

In addition, future work will include a prompt-sensitivity analysis (multiple and optimized prompts), as the present study relies on a single prompt, and a public repository of prompts, generated code, data references, and evaluation scripts.

## Data Availability

The original contributions presented in the study are included in the article/supplementary material, further inquiries can be directed to the corresponding author.
